# A Multi-User Encryption and Authentication System Based on Joint Transform Correlation

**DOI:** 10.3390/e21090850

**Published:** 2019-08-31

**Authors:** Tieyu Zhao, Yingying Chi

**Affiliations:** Information Science Teaching and Research Section, Northeastern University at Qinhuangdao, Qinhuangdao 064000, China

**Keywords:** image encryption, joint transform correlation, optical signal processing

## Abstract

Optical image encryption technology based on joint transform correlation (JTC) has attracted increasing attention from researchers. We propose a multi-user secure communication system based on the special properties of JTC. Multiple users utilize their own fingerprints to encrypt the plain-text in the encryption process, and each user must be first authenticated at the beginning of the decryption process. Only users with legitimate identities can perform the decryption process, whereas unauthorized users with false identities cannot, so the security of the system is greatly improved. Furthermore, we propose a multi-user double-image encryption method, which can better meet the needs of different security environments. Considering the possibility of overlapping images at the output end, we prove that a JTC-based image encryption system can avoid overlapping images at the output when the distance between the input images is 3W (W is the side length of a square image), which provides a theoretical foundation for further research. Finally, a numerical simulation demonstrates the effectiveness and feasibility of the proposed idea.

## 1. Introduction

An optical image encryption method based on joint transform correlation (JTC) is simple and easy to implement and combine with other encryption methods. It has thus become an important branch of optical information security. In 2000, Nomura and Javidi proposed an image encryption system based on JTC [1]. The advantage of its nonlinearity greatly improved the security and reliability of the system. Subsequently, JTC systems have been thoroughly studied and elaborate structures have been proposed [2,3]. Further, Mela and Lemmi introduced an optical encryption technique using three-step phase-shifting interferometry based on JTC in the Fresnel domain [4]. The technique allows a high-speed encryption–decryption process for binary data and is able to operate in a robust and extremely simple optoelectronic system. Soon after, Amaya et al. proposed a multi-channeling encryption method using multiple random-phase mask apertures based on JTC [5,6]. The multiplexing system was immune to known attack procedures because of the existence of an input encryption image pair. Therefore, in order to improve the security of the JTC system, various encryption schemes have been proposed. A multiple phase-shifted encryption method with four parallel channels was proposed by Islam [7]. Alsamman et al. proposed a novel JTC system in which the stored reference image is phase-encrypted prior to applying JTC [8]. The encryption disperses all of the trivial correlation peaks in the correlator output, and the reference image is encrypted electronically, which simplifies the need for a complex optical setup. Meanwhile, the digital holographic configuration was introduced to JTC and the encryption methods were presented with three-dimensional keys [9,10] and a hidden information-encoding architecture [11], and a double-image encryption method using two-step-only quadrature phase-shifting digital holography based on JTC was proposed [12]. Recently, Vilardy et al. analyzed JTC encryption methods and found that it is possible to significantly improve the quality of the decrypted image by introducing a simple nonlinear operation in the encrypted function that contains the joint power spectrum [13,14,15]. Moreover, the JTC system has been applied to different transformation domains, such as the fractional Fourier transform domain [16], Fresnel transform domain [17], and chaotic maps [18,19], which greatly expanded the application of JTC. As is known, cryptography and cryptanalysis are complementary to each other. Therefore, attack schemes against JTC encryption system are proposed [20,21,22,23,24,25,26]. At the same time, the researchers introduced authentication to improve the security of the system [27,28,29,30].

In the JTC encryption system, the common feature is that the classified information is controlled by one user. However, in some cases, this information cannot be owned by one user alone. The classified information can be obtained only if two or more users are present simultaneously. In the current study, a multi-user encryption system is proposed based on JTC. The proposed cryptosystem is uniquely useful in practical applications such as co-signing important documents, the control of nuclear buttons, access to certain crucial information, and so on.

The remainder of this paper is organized as follows. Section 2 proves the feasibility of the proposed scheme through mathematical theory and the analysis of potential risks. Multi-user double image encryption is proposed, and the position distribution of the output image is analyzed in Section 3. Simulation results are shown in Section 4. Finally, the conclusions are presented in Section 5.

## 2. The Proposed Cryptosystem

**Figure 1:**
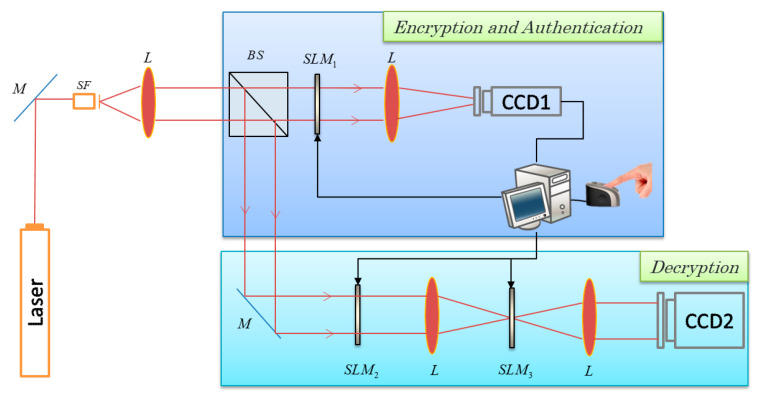
Optical setup. SLM, spatial light modulator. CCD, charge coupled device.

On he basis of JTC, the current report proposes a multi-user encryption system with fingerprint keys. Different system users utilize their own fingerprints to encrypt the same plaintext. The decryption process is flexible because different authorized users can accomplish decryption through varying decryption procedures. The optical setup of the proposed system is shown in Figure 1.

**Figure 2:**
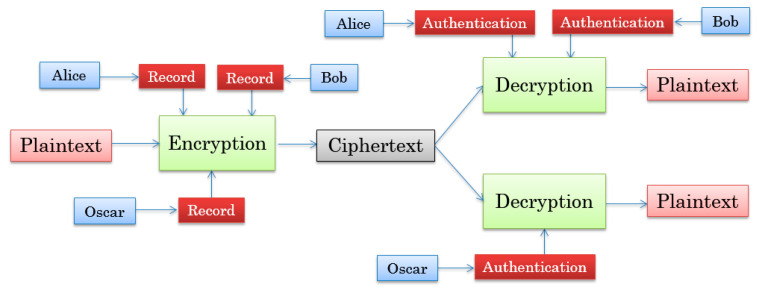
Schematic diagrams.

A two-user encryption system was recently proposed based on JTC [31], which is the special case presented in this paper. An encryption design for three users, as shown in Figure 2, is used as an example to illustrate how the system works.

The detailed process is as follows:

(1) The fingerprints of Alice, Bob, and Oscar are recorded and processed by the computer, respectively, and the encryption keys are obtained.

(2) The plain-text and keys are placed at different locations in the spatial light modulator (SLM) so that the combined transformation power spectrum is obtained as cipher-text.

(3) The users must be authenticated before the decryption. Only authorized users with verified identities can participate in decryption, whereas unauthorized users with uncertified identities cannot conduct the decryption.

(4) The decryption process is flexible. Any authorized user (such as Oscar) can accomplish the decryption themselves. Alternatively, the other two users (Alice and Bob) together can conduct the decryption (each alone cannot complete the decryption).

Assuming that the system has *P* users, considering the need for decryption, users should be grouped before encryption. For example, the first group has *m* users; the second group has *n* users. Here, *P* = *m* + *n*. The requirement for obtaining plain-text is that users not only pass authentication, but also satisfy the specified number of users. For example, the first group must have *m* users appearing at the same time to perform decryption, while the second group must have *n* users appearing at the same time to perform decryption. 

The proposed scheme has a wide range of application scenarios in real life, such as in business or the military; some secrets will require the presence of multiple participants in order to obtain them. Another advantage of the scheme is that there are alternatives, and once a group of users has an accident, another group can be enabled. The flexibility of the scheme is that the number of users in each group can be set according to the decryption requirements.

### 2.1. Fingerprint Recording and Encryption Process

With the rapid development of information technology, the use of human biological features as passwords has been integrated into our lives, such as fingerprint unlocking for mobile phones, face unlocking for computers, and so on. These biological codes are inherent biological characteristics of the human body. Once they are leaked, they will cause immeasurable losses for users [32,33,34]. 

In order to protect user privacy, fingerprints are pre-processed and phase extracted as encryption/decryption keys stored in the computer. The recorded fingerprints ($f_{a}$ is Alice, $f_{b}$ is Bob, and $f_{s}$ is Oscar) are processed by the computer, yielding the keys $k_{a}$, $k_{b}$, and $k_{s}$, respectively.

(1) $$k_{a}=FT\left[f_{a}\right]/\left|FT\left[f_{a}\right]\right|,$$

(2) $$k_{b}=FT\left[f_{b}\right]/\left|FT\left[f_{b}\right]\right|,\;\mathrm{and}$$

(3) $$k_{s}=FT\left[f_{s}\right]/\left|FT\left[f_{s}\right]\right|,$$

where FT denotes the Fourier transform.

**Figure 3:**
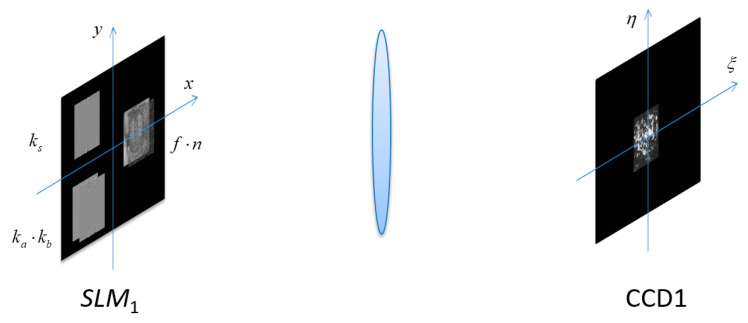
Schematic diagrams of the encryption process.

In Figure 3, the product of the plaintext f(x,y) and the random-phase mask n(x,y) is placed at the coordinate (x=a,y=0). The key $k_{a}\cdot k_{b}$ and the key $k_{s}$ are placed at the coordinates (x=−a,y=−b) and (x=−a,y=b), respectively.

Let

(4) g(x−a,y)=f(x−a,y)⋅n(x−a,y), and

(5) $$k_{\phi }\left(x+a,y+b\right)=k_{a}\left(x+a,y+b\right)\cdot k_{b}\left(x+a,y+b\right).$$

Thus, the joint transform power spectrum is obtained:

(6) $$E\left(\xi ,\eta \right)={\left|FT\left[g\left(x-a,y\right)+k_{s}\left(x+a,y-b\right)+k_{\phi }\left(x+a,y+b\right)\right]\right|}^{2},$$

let

(7) G(ξ,η)=FT[g(x,y)],

(8) $$K_{s}\left(\xi ,\eta \right)=FT\left[k_{s}\left(x,y\right)\right],\;\mathrm{and}$$

(9) $$K_{\phi }\left(\xi ,\eta \right)=FT\left[k_{\phi }\left(x,y\right)\right],$$

so

(10) FT[g(x−a,y)]=G(ξ,η)⋅exp(−j2πaξ),

(11) $$FT\left[k_{s}\left(x+a,y-b\right)\right]=K_{s}\left(\xi ,\eta \right)\cdot \exp\left(j2\pi a\xi \right)\cdot \exp\left(-j2\pi b\eta \right),\;\mathrm{and}$$

(12) $$FT\left[k_{\phi }\left(x+a,y+b\right)\right]=K_{\phi }\left(\xi ,\eta \right)\cdot \exp\left(j2\pi a\xi \right)\cdot \exp\left(j2\pi b\eta \right).$$

Thus, Equation (6) can be written as

(13) $$\begin{array}{cc} E\left(\xi ,\eta \right) & ={\left|G\right|}^{2}+G\cdot K_{s}^{*}\cdot \exp\left(-j4\pi a\xi \right)\exp\left(j2\pi b\eta \right)+G\cdot K_{\phi }^{*}\cdot \exp\left(-j4\pi a\xi \right)\exp\left(-j2\pi b\eta \right)\\ & +K_{s}\cdot G^{*}\cdot \exp\left(j4\pi a\xi \right)\exp\left(-j2\pi b\eta \right)+1+K_{s}\cdot K_{\phi }^{*}\cdot \exp\left(-j4\pi b\eta \right)\\ & +K_{\phi }\cdot G^{*}\cdot \exp\left(j4\pi a\xi \right)\exp\left(j2\pi b\eta \right)+K_{\phi }\cdot K_{s}^{*}\cdot \exp\left(j4\pi b\eta \right)+1. \end{array}$$

From this, we can obtain the cipher-text E(ξ,η).

### 2.2. Authentication

**Figure 4:**
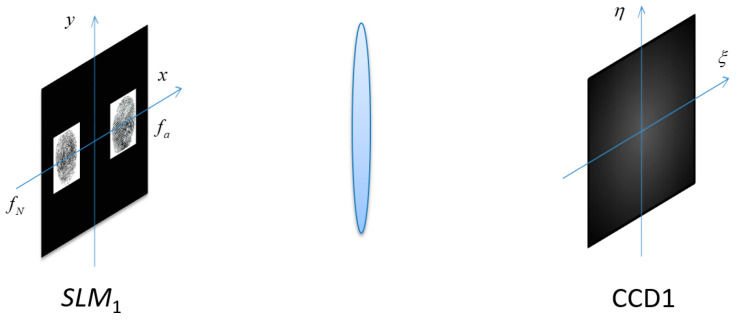
Schematic diagrams of the authentication.

As previously mentioned, the user must first be authenticated before decrypting the cipher-text; that is, the cipher-text can only be decrypted by a user with an authorized identity. After Alice presses her original registered finger on the scanner again, a new fingerprint $f_{N}$ is obtained similar to the original fingerprint $f_{a}$, as shown in Figure 4.

The light intensity distribution is obtained:

(14) $$\begin{array}{cc} O\left(\xi ,\eta \right) & ={\left|FT\left[f_{a}\left(x-a,y\right)+f_{N}\left(x+a,y\right)\right]\right|}^{2}\\ & ={\left|F_{a}\right|}^{2}+{\left|F_{N}\right|}^{2}+F_{a}{}^{*}\cdot F_{N}\exp\left(-j4\pi a\xi \right)+F_{a}\cdot F_{N}^{*}\exp\left(j4\pi a\xi \right). \end{array}$$

The intensity spectrum O(ξ,η) is again put into the input plane and then Fourier transformed:

(15) $$\begin{array}{cc} I\left(x,y\right) & =f_{a}\left(x,y\right)★f_{a}\left(x,y\right)+f_{N}\left(x,y\right)★f_{N}\left(x,y\right)\\ & +f_{a}\left(x,y\right)★f_{N}\left(x,y\right)*\delta \left(x+2a,y\right)+f_{a}\left(x,y\right)★f_{N}\left(x,y\right)*\delta \left(x-2a,y\right). \end{array}$$

where ★ denotes the correlation operation and ∗ denotes the convolution operation. 

**Figure 5:**
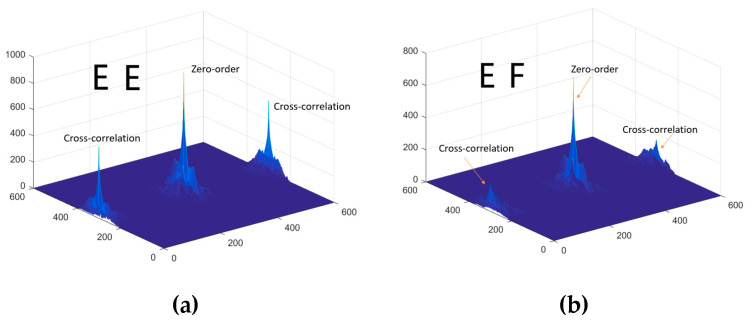
The matching process of letters. (**a**) The same letter, E; (**b**) different letters, E and F.

**Figure 6:**
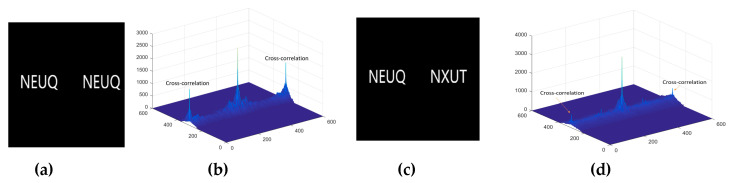
Sensitivity analysis. (**a**) The same letters; (**b**) the peak value of correlation for (**a**); (**c**) different letters; (**d**) the peak value of correlation for (**c**).

Inspired by the works of [35,36], we find that the peak value of cross-correlation affects the results of authentication. The test results are shown in Figure 5 and Figure 6, respectively.

**Figure 7:**
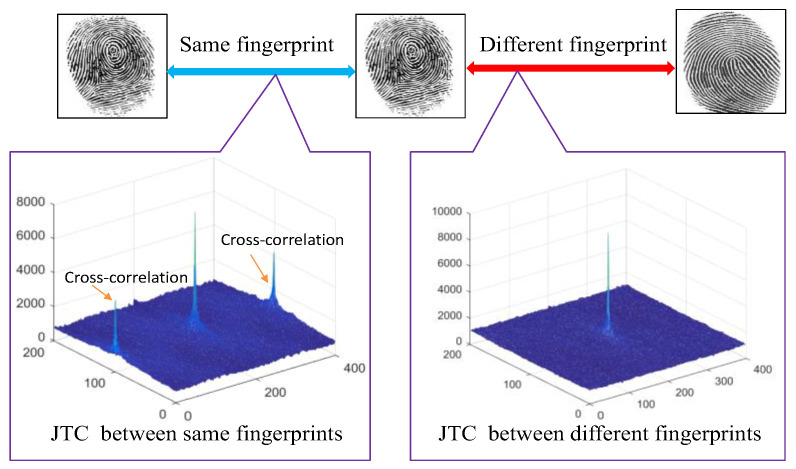
Fingerprint matching. JTC, joint transform correlation.

The test result shows that the matching system based on JTC is very effective. Only an exact match can achieve the maximum peak. The third and fourth terms in Equation (15). show the similarity between the two fingerprints—the left two fingerprints, as shown in Figure 7—while an unauthorized user with an unregistered fingerprint, such as the right image in Figure 7, fails the authentication.

**Figure 8:**
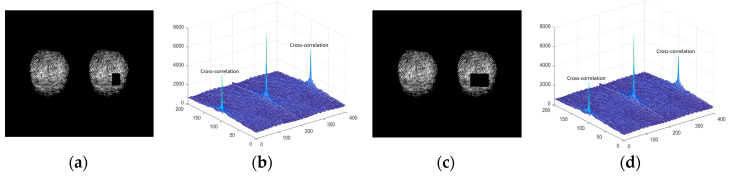
Verification results for locally damaged fingerprints (**a**) The damaged fingerprint; (**b**) the peak value of correlation for (**a**); (**c**) the damaged fingerprint; (**d**) the peak value of correlation for (**c**).

However, we find that the rotation of the fingerprint seriously affects the matching results (as in our recent research work, presented in the work of [31]). When fingerprints are corrupted by noise, it will affect the authentication process. Here, the fingerprint is partially damaged, and the verification results obtained are shown in Figure 8. From the comparison results, we can see that the fingerprint recognition system based on JTC has good robustness against noise attacks.

### 2.3. Decryption Process 

The decryption process is flexible, and two alternative solutions are presented: (1) Oscar conducts the decryption process alone, and (2) Alice and Bob perform the decryption operation together. 

#### 2.3.1. Oscar’s Decryption 

**Figure 9:**
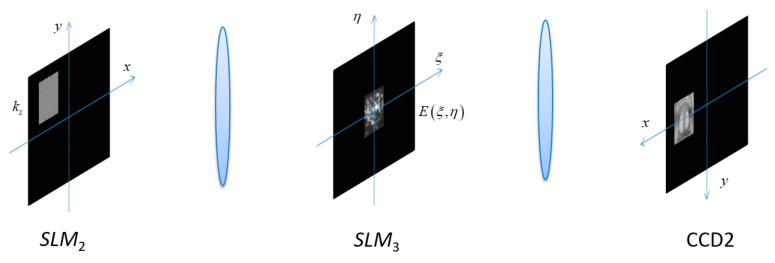
Oscar’s decryption process.

When Oscar passes the authentication, the recorded fingerprint key $k_{s}$ and the cipher-text E(ξ,η) are input into the input plane (SLM2) and the spectrum plane (SLM3), respectively, as shown in Figure 9. 

The detailed process is as follows:

Because

(16) $$FT\left[k_{s}\left(x+a,y-b\right)\right]=K_{s}\cdot \exp\left(j2\pi a\xi \right)\cdot \exp\left(-j2\pi b\eta \right),$$

according to Equation (13), the Fourier spectrum on the front focal plane is

(17) $$\begin{array}{cc} P\left(\xi ,\eta \right) & =E\left(\xi ,\eta \right)\cdot FT\left[k_{s}\left(x+a,y-b\right)\right]\\ & =E\left(\xi ,\eta \right)\cdot K_{s}\cdot \exp\left(j2\pi a\xi \right)\cdot \exp\left(-j2\pi b\eta \right)\\ & ={\left|G\right|}^{2}\cdot K_{s}\cdot \exp\left(j2\pi a\xi \right)\cdot \exp\left(-j2\pi b\eta \right)+G\cdot \exp\left(-j2\pi a\xi \right)\\ & +\left(G\cdot K_{\phi }^{*}\right)\cdot K_{s}\cdot \exp\left(-j2\pi a\xi \right)\cdot \exp\left(-j4\pi b\eta \right)\\ & +\left(K_{s}\cdot G^{*}\right)\cdot K_{s}\cdot \exp\left(j6\pi a\xi \right)\cdot \exp\left(-j4\pi b\eta \right)\\ & +K_{s}\cdot \exp\left(j2\pi a\xi \right)\cdot \exp\left(-j2\pi b\eta \right)\\ & +\left(K_{s}\cdot K_{\phi }^{*}\right)\cdot K_{s}\cdot \exp\left(j2\pi a\xi \right)\cdot \exp\left(-j6\pi b\eta \right)\\ & +\left(K_{\phi }\cdot G^{*}\right)\cdot K_{s}\cdot \exp\left(j6\pi a\xi \right)\\ & +K_{\phi }\cdot \exp\left(j2\pi a\xi \right)\cdot \exp\left(j2\pi b\eta \right)\\ & +K_{s}\cdot \exp\left(j2\pi a\xi \right)\cdot \exp\left(-j2\pi b\eta \right) \end{array}$$

Then, using the inverse Fourier transform,

(18) $$\begin{array}{cc} p\left(x,y\right) & =FT^{-1}\left[P\left(\xi ,\eta \right)\right]\\ & =\left(g★g\right)*k_{s}*\delta \left(x+a,y-b\right)\\ & +g*\delta \left(x-a,y\right)+\left(g★k_{\phi }\right)*k_{s}*\delta \left(x-a,y-2b\right)\\ & +\left(k_{s}★g\right)*k_{s}*\delta \left(x+3a,y-2b\right)+k_{s}*\delta \left(x+a,y-b\right)\\ & +\left(k_{s}★k_{\phi }\right)*k_{s}*\delta \left(x+a,y-3b\right)+\left(k_{\phi }★g\right)*k_{s}*\delta \left(x+3a,y\right)\\ & +k_{\phi }*\delta \left(x+a,y+b\right)+k_{s}*\delta \left(x+a,y-b\right). \end{array}$$

In Equation (18), the plain-text g(x,y)=f(x,y)⋅n(x,y) is obtained using the second term of the sum on the right-hand side of the equation; that is, the plain-text |g(x,y)|=f(x,y) is obtained at coordinates x=a.

#### 2.3.2. Alice and Bob’s Decryption

**Figure 10:**
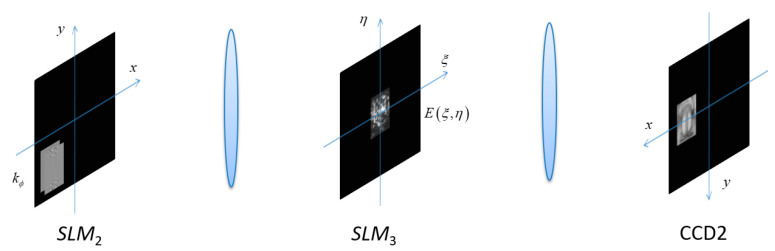
Alice and Bob’s decryption.

Both Alice and Bob must provide their fingerprints together for the decryption, as each alone cannot accomplish the decryption. The decryption process is shown in Figure 10.

According to Equation (13), the Fourier spectrum on the front focal plane is

(19) $$\begin{array}{cc} Q\left(\xi ,\eta \right) & =E\left(\xi ,\eta \right)\cdot FT\left[k_{\phi }\left(x+a,y+b\right)\right]\\ & =E\left(\xi ,\eta \right)\cdot K_{\phi }\cdot \exp\left(j2\pi a\xi \right)\cdot \exp\left(j2\pi b\eta \right)\\ & ={\left|G\right|}^{2}\cdot K_{\phi }\cdot \exp\left(j2\pi a\xi \right)\cdot \exp\left(j2\pi b\eta \right)\\ & +\left(G\cdot K_{s}^{*}\right)\cdot K_{\phi }\cdot \exp\left(-j2\pi a\xi \right)\cdot \exp\left(j4\pi b\eta \right)\\ & +G\cdot \exp\left(-j2\pi a\xi \right)+\left(K_{s}\cdot G^{*}\right)\cdot K_{\phi }\cdot \exp\left(j6\pi a\xi \right)\\ & +K_{\phi }\cdot \exp\left(j2\pi a\xi \right)\cdot \exp\left(j2\pi b\eta \right)\\ & +K_{s}\cdot \exp\left(j2\pi a\xi \right)\cdot \exp\left(-j2\pi b\eta \right)\\ & +\left(K_{\phi }\cdot G^{*}\right)\cdot K_{\phi }\cdot \exp\left(j6\pi a\xi \right)\cdot \exp\left(j4\pi b\eta \right)\\ & +\left(K_{\phi }\cdot K_{s}^{*}\cdot \right)K_{\phi }\cdot \exp\left(j2\pi a\xi \right)\cdot \exp\left(j6\pi b\eta \right)\\ & +K_{\phi }\cdot \exp\left(j2\pi a\xi \right)\cdot \exp\left(j2\pi b\eta \right). \end{array}$$

Using the inverse Fourier transform, we obtain

(20) $$\begin{array}{cc} q\left(x,y\right) & =FT^{-1}\left[Q\left(\xi ,\eta \right)\right]\\ & =\left(g★g\right)*k_{\phi }*\delta \left(x+a,y+b\right)+\left(g★k_{s}\right)*k_{\phi }*\delta \left(x-a,y+2b\right)\\ & +g*\delta \left(x-a,y\right)+\left(k_{s}★g\right)*k_{\phi }*\delta \left(x+3a,y\right)+k_{\phi }*\delta \left(x+a,y+b\right)\\ & +k_{s}*\delta \left(x+a,y-b\right)+\left(k_{\phi }★g\right)*k_{\phi }*\delta \left(x+3a,y+2b\right)\\ & +\left(k_{\phi }★k_{s}\right)*k_{\phi }*\delta \left(x+a,y+3b\right)+k_{\phi }*\delta \left(x+a,y+b\right). \end{array}$$

The plain-text g(x,y)=f(x,y)⋅n(x,y) is obtained using the third term in the sum of Equation (20). Thus, the plain-text |g(x,y)|=f(x,y) is obtained at coordinates x=a.

From Equations (18) and (20), we can determine that the position of the recovered plain-text has not changed. However, at the same time, a security risk was found. In Equation (18), the key $k_{\phi }$ is restored, while in Equation (20), the key $k_{s}$ is restored. Obviously, such a situation is not advisable in information security. Thus, we perform the following further analysis.

## 3. Further Analysis

### 3.1. Double-Image Encryption

The image encryption system based on JTC can achieve multi-image encryption. It does not require the special treatment of plain-text, so it is more convenient than the previously proposed encryption methods [37,38,39]. 

**Figure 11:**
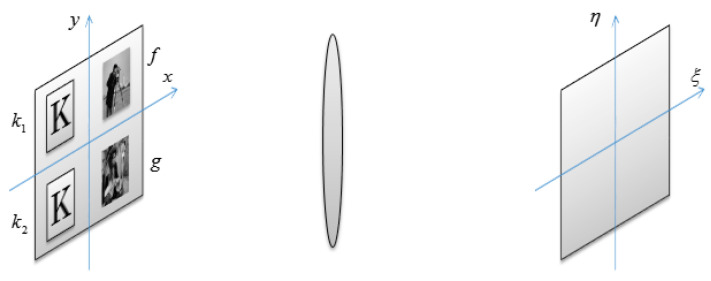
The encryption process diagram.

Here, we use two keys to encrypt two plain-text messages, as shown in Figure 11. Next, we analyze its basic principle. Here, f(x,y) and g(x,y) denote the plain-text, and $k_{1}\left(x,y\right)$ and $k_{2}\left(x,y\right)$ denote the keys. To simplify the proving process, the coordinate position is ignored.

Then, in Figure 11, the cipher-text can be written as

(21) $$\begin{array}{cc} E\left(\xi ,\eta \right) & ={\left|FT\left[f\left(x,y\right)+g\left(x,y\right)+k_{1}\left(x,y\right)+k_{2}\left(x,y\right)\right]\right|}^{2}\\ & ={\left|F\left(\xi ,\eta \right)+G\left(\xi ,\eta \right)+K_{1}\left(\xi ,\eta \right)+K_{2}\left(\xi ,\eta \right)\right|}^{2}\\ & =\left(F+G+K_{1}+K_{2}\right)\left(F^{*}+G^{*}+K_{1}{}^{*}+K_{2}{}^{*}\right). \end{array}$$

When using the key $k_{1}\left(x,y\right)$ for decryption, its expression can be written as

(22) $$\begin{array}{cc} D\left(\xi ,\eta \right) & =E\left(\xi ,\eta \right)\cdot FT\left[k_{1}\left(x,y\right)\right]\\ & =E\left(\xi ,\eta \right)\cdot K_{1}\left(\xi ,\eta \right)\\ & =\left(F+G+K_{1}+K_{2}\right)\left(F^{*}+G^{*}+K_{1}{}^{*}+K_{2}{}^{*}\right)\cdot K_{1}\\ & =\left(F+G+K_{1}+K_{2}\right)\left(F^{*}\cdot K_{1}+G^{*}\cdot K_{1}+K_{1}^{*}\cdot K_{1}+K_{2}{}^{*}\cdot K_{1}\right)\\ & =\left(F+G+K_{1}+K_{2}\right)\left(F^{*}\cdot K_{1}+G^{*}\cdot K_{1}+1+K_{2}{}^{*}\cdot K_{1}\right)\\ & =\left(F+G+K_{1}+K_{2}\right)\left(F^{*}\cdot K_{1}+G^{*}\cdot K_{1}+K_{2}{}^{*}\cdot K_{1}\right)+\left(F+G+K_{1}+K_{2}\right). \end{array}$$

Using the inverse Fourier transform, we obtain

(23) $$\begin{array}{cc} d\left(x,y\right) & =FT^{-1}\left[D\left(\xi ,\eta \right)\right]\\ & =FT^{-1}\left[\left(F+G+K_{1}+K_{2}\right)\left(F^{*}\cdot K_{1}+G^{*}\cdot K_{1}+K_{2}{}^{*}\cdot K_{1}\right)\right]+\\ & f\left(x,y\right)+g\left(x,y\right)+k_{1}\left(x,y\right)+k_{2}\left(x,y\right). \end{array}$$

Equation (23) is solved, and its complete decryption result is obtained as Equation (24).

(24) $$\begin{array}{cc} d\left(x,y\right) & =FT^{-1}\left[D\left(\xi ,\eta \right)\right]\\ & =FT^{-1}\left[\left(F+G+K_{1}+K_{2}\right)\left(F^{*}\cdot K_{1}+G^{*}\cdot K_{1}+K_{2}{}^{*}\cdot K_{1}\right)\right]+f\left(x,y\right)+g\left(x,y\right)+k_{1}\left(x,y\right)+k_{2}\left(x,y\right).\\ & =\left(f★f\right)*k_{1}*\delta \left(x+a,y-b\right)+\left(f★g\right)*k_{1}*\delta \left(x+a,y-3b\right)+\left(f★k_{2}\right)*k_{1}*\delta \left(x-a,y-3b\right)\\ & +\left(g★f\right)*k_{1}*\delta \left(x+a,y+b\right)+\left(g★g\right)*k_{1}*\delta \left(x+a,y-b\right)+\left(g★k_{2}\right)*k_{1}*\delta \left(x-a,y-b\right)\\ & +\left(k_{1}★f\right)*k_{1}*\delta \left(x+3a,y-b\right)+\left(k_{1}★g\right)*k_{1}*\delta \left(x+3a,y-3b\right)+\left(k_{1}★k_{2}\right)*k_{1}*\delta \left(x+a,y-3b\right)\\ & +\left(k_{2}★f\right)*k_{1}*\delta \left(x+3a,y+b\right)+\left(k_{2}★g\right)*k_{1}*\delta \left(x+3a,y+b\right)+k_{1}*\delta \left(x+a,y-b\right)\\ & +f*\delta \left(x-a,y-b\right)+g*\delta \left(x-a,y+b\right)+k_{1}*\delta \left(x+a,y-b\right)+k_{2}*\delta \left(x+a,y+b\right). \end{array}$$

When the key $k_{2}$ is used for decryption, the result of the decryption is as in Equation (25).

(25) $$\begin{array}{cc} d\left(x,y\right) & =FT^{-1}\left[D\left(\xi ,\eta \right)\right]\\ & =FT^{-1}\left[\left(F+G+K_{1}+K_{2}\right)\left(F^{*}\cdot K_{2}+G^{*}\cdot K_{2}+K_{1}{}^{*}\cdot K_{2}\right)\right]+f\left(x,y\right)+g\left(x,y\right)+k_{1}\left(x,y\right)+k_{2}\left(x,y\right).\\ & =\left(f★f\right)*k_{2}*\delta \left(x+a,y+b\right)+\left(f★g\right)*k_{2}*\delta \left(x+a,y-b\right)+\left(f★k_{1}\right)*k_{2}*\delta \left(x-a,y+b\right)\\ & +\left(g★f\right)*k_{2}*\delta \left(x+a,y+3b\right)+\left(g★g\right)*k_{2}*\delta \left(x+a,y+b\right)+\left(g★k_{1}\right)*k_{2}*\delta \left(x-a,y+3b\right)\\ & +\left(k_{1}★f\right)*k_{2}*\delta \left(x+3a,y+b\right)+\left(k_{1}★g\right)*k_{2}*\delta \left(x+3a,y-b\right)+k_{2}*\delta \left(x+a,y+b\right)\\ & +\left(k_{2}★f\right)*k_{2}*\delta \left(x+3a,y+3b\right)+\left(k_{2}★g\right)*k_{2}*\delta \left(x+3a,y+b\right)+\left(k_{2}★k_{1}\right)*k_{2}*\delta \left(x+a,y+3b\right)\\ & +f*\delta \left(x-a,y-b\right)+g*\delta \left(x-a,y+b\right)+k_{1}*\delta \left(x+a,y-b\right)+k_{2}*\delta \left(x+a,y+b\right). \end{array}$$

In Equation (24), we notice that when the key $k_{1}$ is used for decryption, only the plain-text g(x,y) is obtained. We are unable to obtain other information because it is overlapped. Some attack schemes want to extract information from cipher-text, which will be very difficult in reality [22]. Similarly, when decrypting with the key $k_{2}$, we can only obtain the plain-text f(x,y), as shown in Equation (25).

Further, the keys $k_{1}$ and $k_{2}$ can be combined, such as $k_{1}=\phi_{1}\cdot \phi_{2}\cdots \phi_{m}$ and $k_{2}=\varphi_{1}\cdot \varphi_{2}\cdots \varphi_{n}$. Thus, $k_{1}$ means that there are *m* users (Alice, Tom, ……, Emma), and $k_{2}$ means *n* users (Olivia, Hannah, ……, John). Thus, it can be decrypted only when m users appear simultaneously or n users appear simultaneously. Of course, in the keys $k_{1}$ and $k_{2}$, there can be the same key (user); in other words, the user can obtain both the plain-text g and plain-text f.

Furthermore, the proposed scheme does not need to pre-process the plain-text and realizes double image encryption in a real sense compared with other research methods [37,38,39]. In the process of decryption, each group of users can only obtain one plain-text. For example, the first group of users (Alice, Tom, ……, Emma) can only obtain plain-text *g*, and the second group of users (Olivia, Hannah, ……, John) can only obtain plain-text *f*. If a user is in both groups, he/she has the right to access two plain-texts.

In some scenarios, secrets not only need to be shared by multiple users, but also have hierarchical relationships among users [40]. For example, in a company, managers usually have more resources than employees; in the army, officers usually have more information than soldiers. At this time, the traditional cryptosystem we know is insufficient for this, and the scheme proposed in this section has significant advantages.

In Equations (24) and (25), the plain-text messages are restored during decryption; at this time, the position of the restored image is particularly important. Here, we analyze and discuss the circumstances that can ensure that the output images are separated from each other.

### 3.2. Image Position Distribution on Output

Researchers have previously discovered that the positional distribution of the input image affects the distance between the output spots based on JTC’s identification system [41,42,43]. Assuming that the edge length of the given input object image and reference image are both *W*, when the distance between the two images is greater than 2*W*, the output images are separated from each other.

Here, $g\left(x-a_{1},y\right)$ and $r\left(x-a_{2},y\right)$ denote the object image and the reference image, respectively. The light intensity of the output can be expressed as

(26) $$\begin{array}{cc} E\left(\xi ,\eta \right) & ={\left|FT\left[g\left(x-a_{1},y\right)+r\left(x-a_{2},y\right)\right]\right|}^{2}\\ & =\left[G\left(\xi ,\eta \right)\exp\left(-j2\pi a_{1}\xi \right)+R\left(\xi ,\eta \right)\exp\left(-j2\pi a_{2}\xi \right)\right]\cdot \\ & \left[G^{*}\left(\xi ,\eta \right)\exp\left(j2\pi a_{1}\xi \right)+R^{*}\left(\xi ,\eta \right)\exp\left(j2\pi a_{2}\xi \right)\right]\\ & ={\left|G\right|}^{2}+{\left|R\right|}^{2}+G\cdot R^{*}\exp\left(j2\pi \left(a_{2}-a_{1}\right)\xi \right)+R\cdot G^{*}\cdot \exp\left(j2\pi \left(a_{1}-a_{2}\right)\xi \right). \end{array}$$

Using the inverse Fourier transform, we obtain

(27) $$\begin{array}{cc} I\left(x,y\right) & =IFT\left[E\left(\xi ,\eta \right)\right]\\ & =g★g*\delta \left(x,y\right)+r★r*\delta \left(x,y\right)\\ & +g★r*\delta \left(x+\left(a_{2}-a_{1}\right),y\right)+g★r*\delta \left(x+\left(a_{1}-a_{2}\right),y\right). \end{array}$$

**Figure 12:**
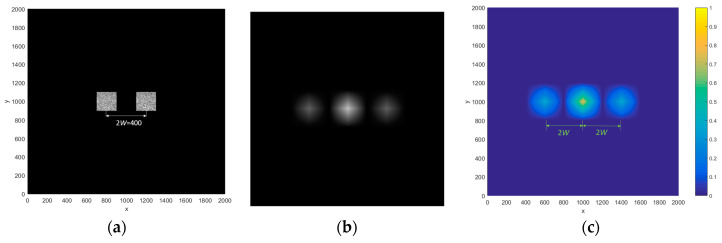
(**a**) The input image, (**b**) the light intensity distribution at the output end, and (**c**) the energy distribution of (**b**).

In Equation (27), four items are obtained. Two items are at the coordinate origin, and the other two are at the two sides of the coordinate origin, as shown in Figure 12. When the distance between the input images is 2W, the output images are guaranteed to be separated from each other; thus, $\left|a_{1}-a_{2}\right|\geq 2W$ (note that the input images’ size is 200 × 200 pixels, so W = 200).

An analysis of the image encryption system based on JTC indicates that the distance between the plain-text and the key is 3W at the input, which can ensure that the output images are separated from each other. The detailed proof is as follows.

Here, $f\left(x-a_{1},y\right)$ and $k\left(x-a_{2},y\right)$ denote the plain-text and the key, respectively, so the cipher-text is obtained in the encryption system and its expression can be written as

(28) $$\begin{array}{cc} E\left(\xi ,\eta \right) & ={\left|FT\left[f\left(x-a_{1},y\right)+k\left(x-a_{2},y\right)\right]\right|}^{2}\\ & ={\left|F\right|}^{2}+{\left|K\right|}^{2}+F\cdot K^{*}\exp\left(j2\pi \left(a_{2}-a_{1}\right)\xi \right)+K\cdot F^{*}\cdot \exp\left(j2\pi \left(a_{1}-a_{2}\right)\xi \right). \end{array}$$

When using the key $k\left(x-a_{2},y\right)$ for decryption, the decryption process can be expressed as

(29) $$\begin{array}{cc} D\left(\xi ,\eta \right) & =E\left(\xi ,\eta \right)\cdot FT\left[k\left(x-a_{2},y\right)\right]\\ & =E\left(\xi ,\eta \right)\cdot K\left(\xi ,\eta \right)\exp\left(-j2\pi a_{1}\xi \right)\\ & =[{\left|F\right|}^{2}+{\left|K\right|}^{2}+F\cdot K^{*}\exp\left(j2\pi \left(a_{2}-a_{1}\right)\xi \right)\\ & +K\cdot F^{*}\cdot \exp\left(j2\pi \left(a_{1}-a_{2}\right)\xi \right)]\cdot K\left(\xi ,\eta \right)\exp\left(-j2\pi a_{2}\xi \right). \end{array}$$

Using the inverse Fourier transform, we obtain

(30) $$\begin{array}{cc} d\left(x,y\right) & =FT^{-1}\left[D\left(\xi ,\eta \right)\right]\\ & =f★f*k*\delta \left(x-a_{2},y\right)+k★k*k*\delta \left(x-a_{2},y\right)\\ & \;+f*\delta \left(x-a_{1},y\right)+k★f*k*\delta \left(x+a_{1}-2a_{2},y\right). \end{array}$$

As demonstrated by Equation (30), each form is unified as $z_{1}\left(x\right)★z_{2}\left(x\right)*z_{3}\left(x\right)$.

Because

(31) $$z_{1}\left(x\right)*z_{2}\left(x\right)*z_{3}\left(x\right)=\int_{-\infty }^{\infty }\left[\int_{-\infty }^{\infty }z_{1}\left(\lambda \right)z_{2}\left(\tau -\lambda \right)d\lambda \right]z_{3}\left(x-\tau \right)d\tau ,$$

(32) $$z_{1}\left(x\right)★z_{2}\left(x\right)*z_{3}\left(x\right)=z_{1}\left(x\right)*z_{2}\left(-x\right)*z_{3}\left(x\right).$$

When the distance between the images is greater than 3W at the input end, the images are mutually separated at the output end.

**Figure 13:**
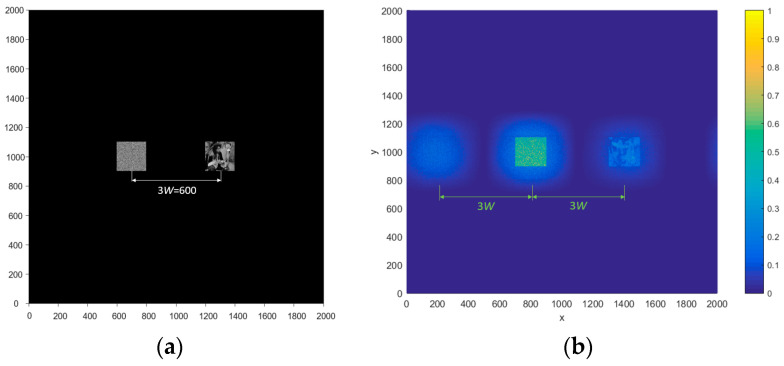
(**a**) The plain-text and key and (**b**) the intensity distribution after decryption.

In Equation (30), the coordinate position is $\left(a_{1},0\right)$, $\left(a_{2},0\right)$, and $\left(2a_{2}-a_{1},0\right)$, respectively. When $\left|a_{1}-a_{2}\right|\geq 3W$, we obtain $\left|\left(2a_{2}-a_{1}\right)-a_{2}\right|=\left|a_{2}-a_{1}\right|\geq 3W$ and $\left|\left(2a_{2}-a_{1}\right)-a_{1}\right|=2\left|\left(a_{2}-a_{1}\right)\right|\geq 6W$, so that the output images are separated from each other, as shown in Figure 13. Here, the input images’ size is 200 × 200 pixels, so *W* = 200.

For JTC’s image encryption system, the output image is $z_{1}\left(x_{i_{1}},y_{j_{1}}\right)★z_{2}\left(x_{i_{2}},y_{j_{2}}\right)*z_{3}\left(x_{i_{3}},y_{j_{3}}\right)$ and does not depend on the number of input images. Therefore, the distance between the input images is either $\left|x_{i}-x_{j}\right|\geq 3W$ or $\left|y_{i}-y_{j}\right|\geq 3W$, which ensures that the output images are separated.

## 4. Numerical Simulation

**Figure 14:**
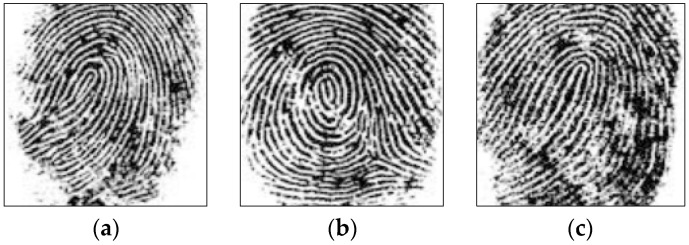

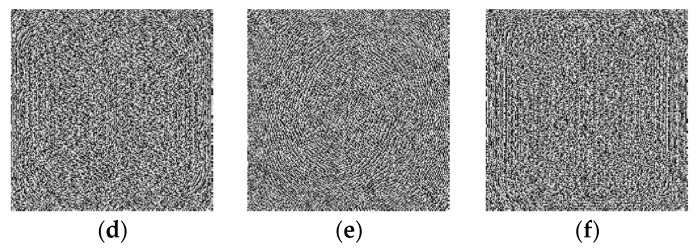
(**a**) Alice’s fingerprint, (**b**) Bob’s fingerprint, (**c**) Oscar’s fingerprint, (**d**) the phase of Alice’s fingerprint, (**e**) the phase of Bob’s fingerprint, and (**f**) the phase of Oscar’s fingerprint.

The fingerprints (Alice, Bob, and Oscar) are shown in Figure 14a–c, respectively, and the phase of each fingerprint is used as a key, as demonstrated in Figure 14d–f.

**Figure 15:**
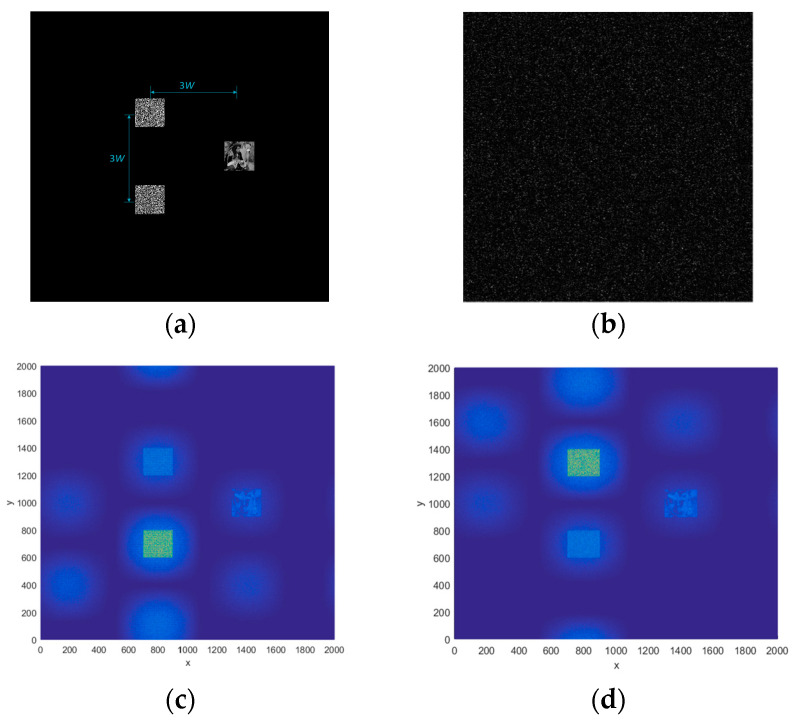
(**a**) The plain image and the keys, (**b**) the cipher-text, (**c**) the decrypted result by Oscar, and (**d**) the decrypted result by Alice and Bob together.

We perform experimental simulations for the proposed cryptosystem, as shown in Figure 15. In Figure 15a, the images’ size is 200 × 200 pixels (*W* = 200). The vertical distance between the two keys at the input is 600, and the horizontal distance between the two keys and the plain-text is also 600. This ensures that the images at the output are independent of each other.

The plain image and the keys are placed at different positions on the input, respectively, as shown in Figure 15a. Here, the keys are on the left (above is Oscar’s key, below is the product of Alice and Bob’s key), and the plain image is on the right. The cipher-text is obtained as shown in Figure 15b. It should be noted that the size of the cipher-text is the same as that of the input surface (2000 × 2000 pixels). The result that Oscar decrypts alone is shown in Figure 15c, and the result that Alice and Bob decrypt together is shown in Figure 15d. 

**Figure 16:**
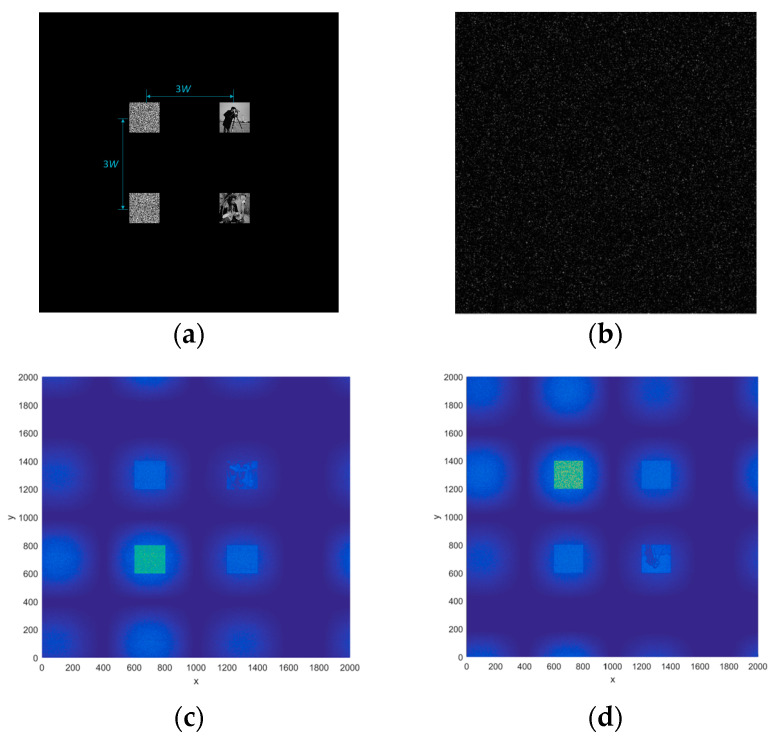
(**a**) The plain images and the keys, (**b**) the cipher-text, (**c**) the decrypted result by Oscar, and (**d**) the decrypted result by Alice and Bob together.

Next, the multi-user double image encryption scheme is analyzed. The keys are still used in Figure 14, so the distribution of the keys and plain images are shown in Figure 16a. Here, the keys are on the left (above is Oscar’s key, below is the product of Alice and Bob’s key), and the two plain-text images are on the right. In Figure 16b, the ciphertext is obtained (2000 × 2000 pixels). The result that Oscar’s decrypt alone is shown in Figure 16c, and the result that Alice and Bob’s decrypt together is shown in Figure 16d. Here, Figure 16c corresponds to Equation (24), because the same coordinate positions are merged, so we see that there are nine items. Similarly, Figure 16d corresponds to Equation (25), and there are also nine items. 

Correlation is an important method to evaluate whether the system can resist statistical analysis attack. For a good encryption method, the cipher-text obtained should be fully confused; that is, the attacker cannot get any plain-text information from the cipher-text.

**Figure 17:**
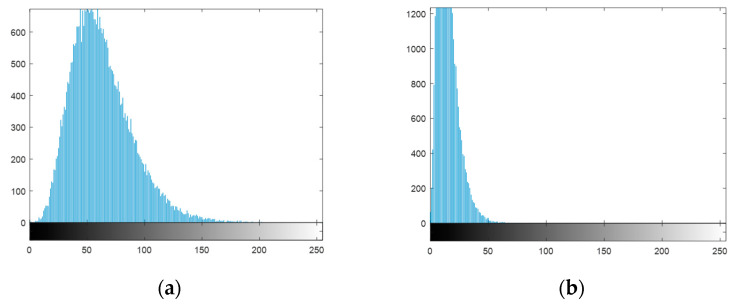
Histogram of the cipher-text: (**a**) histogram of Figure 15b; (**b**) histogram of Figure 16b.

Figure 17 is the histogram of the ciphertext of the two encryption schemes (Figure 15b and Figure 16b, respectively). The cipher-text obtained by the proposed scheme is the energy spectrum. From the histogram, the proposed scheme does not achieve the effect of chaotic encryption [44,45]. 

The impact of noise on the system should mainly come from two aspects: on the one hand, the impact of the fingerprint recognition; on the other hand, the cipher-text will be attacked by noise, which will affect the results of decryption [46].

**Figure 18:**
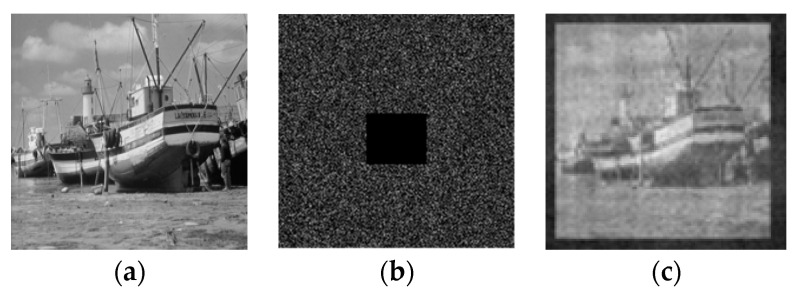
(**a**) The plain-text; (**b**) the cipher-text; (**c**) the decrypted image.

When fingerprints are corrupted by noise, it will affect the authentication process. Here, the fingerprint is partially damaged, and the verification results obtained are shown in Figure 3. The decryption process is built in the optical system. Owing to the optical holography, the system is robust against interference. When the ciphertext is partially damaged, the decryption results are shown in Figure 18. Here, we give the mean square error and the correlation coefficient (*MSE* = 52; *CC* = 0.7538).

The simulation analysis not only verifies the effectiveness of the proposed scheme, but also proves that the image encryption system based on JTC can ensure that the output image cannot be overlapped when the distance between the input images is three times the edge length of the image. In the performance analysis, the system shows good anti-jamming ability. However, the obtained cipher-text is an energy spectrum, and its encryption process is not ideal for plain-text confusion.

## 5. Conclusions

This paper proposed a multi-user authentication image encryption scheme based on JTC. A user’s fingerprint can not only be used as a key, but can also be authenticated. In order to protect user privacy, fingerprints are pre-processed and phase extracted as encryption/decryption keys stored in the computer. In the proposed scheme, two conditions must be met for the plain-text to be successfully restored: (1) before decryption, the user must be authenticated, and only those with legitimate identities can participate in decryption; and (2) each group of users must reach a specified number of users before they can be decrypted (users are grouped before encryption). As far as we know, this scheme is not involved in previous encryption methods. The feasibility and effectiveness of the proposed scheme were verified by mathematical derivation and numerical simulation. However, with the deepening of the research, we found that the scheme has the hidden danger of key leakage in the decryption process. Therefore, we further proposed a multi-user double image encryption scheme to overcome the hidden danger. The new scheme not only satisfies the above two security conditions, but also adds a hierarchical relationship between users. Only specific users have the right to access two images, while other users can only access one of them. At this point, considering the position of the restored image is particularly important. We analyzed and discussed the circumstances that can ensure that the output images are separated from each other. Finally, the performance of the proposed scheme is analyzed by numerical simulation.

In the future, we will study multi-user color image encryption, because color images contain more information [44]. At present, our research only applies to gray images. At the same time, the positions of the input images are symmetrical. If the positions of the images are irregularly arranged, the correlation between the output images will need to be further studied. As a stage study, this paper hopes to give some inspiration to researchers.
